# Molecular Detection of Feline Coronavirus in Captive Non-Domestic Felids from Zoological Facilities

**DOI:** 10.3390/ani12141864

**Published:** 2022-07-21

**Authors:** Gabriele Ratti, Angelica Stranieri, Alessia Giordano, Maurizio Oltolina, Eleonora Bonacina, William Magnone, Manuel Morici, Giuliano Ravasio, Saverio Paltrinieri, Stefania Lauzi

**Affiliations:** 1 Department of Veterinary Medicine and Animal Science, University of Milan, Via dell’Università 6, 26900 Lodi, Italy; gabriele.ratti@unimi.it (G.R.); angelica.stranieri@unimi.it (A.S.); giuliano.ravasio@unimi.it (G.R.); saverio.paltrinieri@unimi.it (S.P.); stefania.lauzi@unimi.it (S.L.); 2 Parco Faunistico Le Cornelle, Via Cornelle 16, 24030 Valbrembo, Italy; oltolina@lecornelle.it; 3 Ambulatorio Veterinario San Rocco, Viale della Vittoria 23/A, 23815 Introbio Valsassina, Italy; eleonorabonacina@libero.it; 4 Parco Natura Viva Garda Zoological Park S.r.l, Località Quercia, 37012 Bussolengo, Italy; william.magnone@parconaturaviva.it; 5 Pombia Park S.r.l., Via Larino 3, 28050 Pombia, Italy; manuel.morici@safaripark.it

**Keywords:** feline coronavirus, tiger, zoo, RT-qPCR

## Abstract

**Simple Summary:**

Non-domestic felids are well-known threatened species, and are susceptible to several diseases that also affect domestic cats. Among viral infections, fatal outbreaks of feline infectious peritonitis, caused by a feline coronavirus, have been reported in captive settings. Considering the devastating effects that this pathogen could have in non-domestic felids, the aim of this study was to assess the prevalence of feline coronavirus in captive non-domestic felids from Northern Italy, since in the literature, this information is not currently available. The overall prevalence of feline coronavirus in captive non-domestic felids from Northern Italy was 7.9%. Results of the present study highlight the need of control programs for feline coronavirus infection to prevent pathogen introduction into a naïve group of animals, which may lead to devastating effects on animal welfare and conservation programs.

**Abstract:**

Cases of feline infectious peritonitis (FIP), a disease with a high mortality rate caused by the feline coronavirus (FCoV), have been reported in non-domestic felids, highlighting the need for surveys of FCoV in these endangered species. With the aim of adding information on FCoV prevalence in captive non-domestic felids, samples (feces or rectal swabs and, when available, oral swabs, blood, and abdominal effusion) collected between 2019 and 2021 from 38 non-domestic felids from three different zoological facilities of Northern Italy were tested for evidence of FCoV infection via RT-qPCR. Three animals were found to be FCoV positive, showing an overall 7.9% FCoV prevalence ranging from 0% to 60%, according to the zoological facility. FCoV infection was detected in tiger cubs of the same litter, and all of them showed FCoV-positive oral swabs, with low viral loads, whereas in one animal, FCoV presence was also detected in rectal swabs at low FCoV copy numbers. Future studies should be carried out, including samplings from a higher number of captive non-domestic felids, in order to gain a deeper knowledge of FCoV epidemiology within these populations.

## 1. Introduction

Infectious diseases can have a great impact on the welfare and health of wildlife, and can also adversely affect conservation plans that aim to protect endangered species [1,2]. Moreover, infectious diseases have been identified as one of the most common causes of mortality in animals housed in zoos, and this has also been observed recently in Italy [3]. Non-domestic felids are well-known threatened species and are susceptible to several diseases that also affect domestic cats [4,5,6]. Among viral infections, worldwide attention has recently focused on the severe acute respiratory syndrome coronavirus (SARS-CoV-2), belonging to the betacoronavirus genus, which is the cause of the COVID-19 pandemic [2]. However, other coronaviruses have been frequently reported to infect felids, such as feline coronavirus (FCoV), which belongs to the alphacoronavirus genus, with worldwide distribution in cats. The presence of FCoV has been investigated in non-domestic felids housed in zoological facilities and in wildlife showing variable FCoV prevalence rates [5,7,8,9,10,11,12,13]. FCoV infection is often subclinical but clinical diseases, ranging from mild enteritis to fatal feline infectious peritonitis (FIP), have been observed in both domestic and non-domestic felids [6,14,15,16]. 

Studies using serological and molecular tests have mainly investigated cheetahs (*Acinonyx jubatus*), reporting FCoV high positivity rates and FIP cases [4,7,11,14,17]. Regarding other non-domestic felids, FCoV infection and FIP cases have been reported in African lions (*Panthera leo*), Tigers (*Panthera tigris*), Mountain lions (*Puma concolor*), European wild cats (*Felis silvestris*), Servals (*Leptailurus serval*), Bobcats (*Lynx rufus*), Jaguars (*Panthera onca*), Leopards (*Panthera pardus*), Pallas cats (*Otocolobus manul*), and Sand cats (*Felis margarita*) [2,18]. Moreover, FCoV infection in the absence of credible reports of FIP cases has been reported in in several non-domestic felids other than cheetahs, including Asian leopard cats (*Prionailurus bengalensis*), Ocelots (*Leopardus pardalis*), Margay cats (*Leopardus wiedii*), Geoffroy’s cats (*Leopardus geoffroyi*), Caracals (*Caracal caracal*), Snow leopards (*Panthera uncia*), Iberian lynx (*Lynx pardinus*), and Canadian lynx (*Lynx canadensis*) [2,10]. 

Most cases of FIP outbreaks in non-domestic felids have been reported in captive settings [19,20,21,22], but a case of FIP has also been observed in a free-living Mountain lion [23]. One of the most severe FIP outbreaks recorded in either domestic cats or non-domestic felids was reported in cheetahs in 1982–1987 at a zoo in Oregon, in which a total of 27 cheetahs died of FIP, whereas 18 animals exposed to FCoV survived, corresponding to 60% mortality [19,20]. Introduction into a naïve population, captivity-induced stress, and environmental factors in cheetahs may be significant in FIP pathogenesis [24,25,26]. A significant higher mortality rate in young compared to subadult and adult animals was observed during the FIP 1982–1987 outbreak in cheetahs [20]. Similarly, environmental factors, stress, and young age are known to be associated with FIP in cats [15,18]. 

Due to the worldwide presence of FCoV in domestic cats, and the cases of FIP that have been reported in non-domestic felids, surveys of FCoV in populations of non-domestic felids have been recommended to prevent the devastating effects of FCoV introduction into a naïve group of animals [2]. To the best of our knowledge, studies investigating the presence of FCoV in captive felids from European zoological collections have been rarely reported [21]. Therefore, the present study aims to investigate the presence of FCoV in non-domestic felids from different zoological facilities in Northern Italy through RT-qPCR analysis.

## 2. Materials and Methods

### 2.1. Animals and Samples Collection

Animals from zoological facilities belonging to the Felidae family that were admitted to the Veterinary Teaching Hospital (VTH) of Lodi, University of Milan, Italy, for diagnostic purposes were included in this study under informed consensus from the veterinarians of the zoological facilities that housed the animals. 

Samples were collected during the period June 2019–July 2021. Feces were collected within 12 h of deposition, directly from the facility that hosted the animals or from transport cages, if animals had undergone diagnostic investigations at the VTH, and were placed in suitable containers. Rectal swabs and oral swabs were either collected from the animals from the zoological facility or from pharmacologically immobilized animals during the diagnostic investigations carried out at the VTH. Blood and abdominal effusion samples were collected only from pharmacologically immobilized animals during the diagnostic investigations carried out at the VTH. Fecal samples, rectal swabs, and oral swabs were stored at 4 °C until arrival at the laboratory (within 12 h), and then stored at −80 °C until RNA extraction. Aliquots of blood and abdominal effusion samples, when present, were placed in tubes containing EDTA and stored at −80 °C until molecular analysis. According to the Ethical Committee decision of the University of Milan, residual aliquots of samples or tissues collected for diagnostic purposes at the VTH under informed consent of the owners can be used for research purposes without any additional formal request of authorization (EC decision 29 October 2012, renewed with the protocol no. 02-2016). 

For each animal, information regarding the signalment (species, sex, age, and zoological collection of origin) clinical status, and definitive diagnosis based on diagnostic activity carried out in VTH was collected. The age variable was divided into two categories: ≤2 years old and >2 years old. The clinical status variable was divided into three categories: clinically healthy animals, animals with clinical signs possibly suggestive of FIP (abdominal effusions, central nervous system, and ocular abnormalities), and diseased animals with clinical signs not suggestive of FIP [18].

The total number of non-domestic felids housed in zoological facilities in Italy and in Northern Italy was assessed using Zoological Information Management System (http://zims.Species360.org, accessed on 20 January 2022).

### 2.2. Real-Time Reverse Transcriptase PCR (RT-qPCR)

RNA extraction from samples was performed using a commercial NucleoSpin viral RNA isolation kit or NucleoSpin RNA isolation kit (Macherey-Nagel, Bethlehem, PA, USA), according to the type of sample, following the manufacturer’s instructions.

RNA quality control targeting vertebrate 12S rRNA locus [27] was performed on randomly selected samples (results not shown). Extracted RNA was subjected to RT-qPCR, based on the amplification of the 7b gene of FCoV, as previously described [28]. The RT-qPCR reaction was performed using a QS3 instrument (Applied Biosystems). Briefly, the RT-qPCR reaction was set up using a commercial kit (TaqMan Fast Virus 1step master mix, Applied Biosystems) in a final volume of 25 µL, with minor modifications. The following primers and probe were used: (Forward primer FCoV 1128f: 5′-GAT TTG ATT TGG CAA TGC TAG ATT T-3′; Reverse primer FCoV 1229r: 5′-ACC AAT CAC TAG ATC CAG ACG TTA GCT-3′; FCoV1200p: FAM-5′ TCC ATT GTT GGC TCG TCA TAG CGG A-3′-TAMRA). After a reverse transcription phase of 5 min at 50 °C and activation of 20 s at 95 °C, 40 cycles with following condition were used: 3 s at 95 °C and 1 min at 60 °C. As a positive control, a FCoV-positive cat sample was used, represented by RNA extracted from the spleen of a cat diagnosed with FIP. The negative control consisted of a FCoV-negative sample from a domestic cat, and a blank control (RNase-free water sample) was also included in all RT-qPCR reactions. A sample was considered positive in the presence of an amplification curve and a value of threshold cycles (Ct) < 40, as previously reported [29]. For absolute quantitation, serial log_10_ dilutions of a pCR4 plasmid (Invitrogen, Carlsbad, CA, USA) containing the FCoV 7b target sequence with a known copy number (10^1^–10^7^ copies/µL), produced according to previously published protocols [30] and kindly provided by Professor Mara Battilani, were amplified with the samples in order to obtain a standard curve.

Animals with a positive RT-qPCR result in at least one sample were considered FCoV positive.

## 3. Results

**Figure 1:**
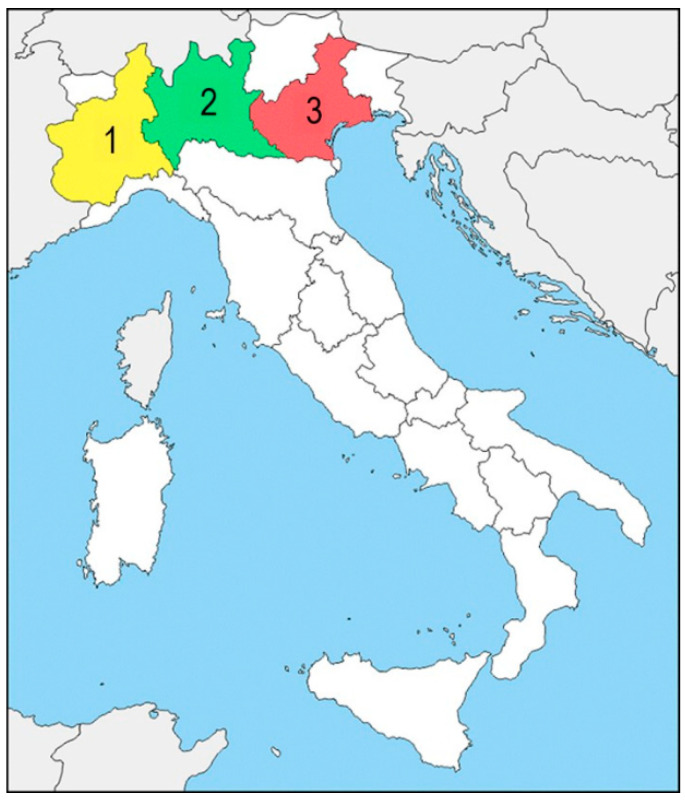
Geographic localization of the zoological facilities from Northern Italy included in this study. 1: Zoological facility from Piedmont; 2: zoological facility from Lombardy; 3: zoological facility from Veneto.

Thirty-eight animals belonging to the Felidae family were sampled. Animals were from three zoological collections from Northern Italy, located in the Piedmont, Lombardy, and Veneto regions, respectively (Figure 1).

Considering that 166 non-domestic felids have been reported by the Zoological Information Management System to be housed in Italy and 114 have been reported in Northern Italy, the 38 animals sampled represent 22.9% and 33% of the total Italian and the Northern Italian population of non-domestic felids, respectively. The characteristics of the sampled animals are reported in Table 1. The number and characteristics of collected samples are reported in Table 2. Feces or rectal swabs were collected from all animals. The same day of feces or rectal swabs collection, oral swabs were collected from three animals, blood samples were collected from four animals, and abdominal effusion samples were collected from two animals. A total of 60 samples were collected, consisting of 50 fecal samples (41 feces and 9 rectal swabs), 4 whole blood samples, 2 abdominal effusion samples, and 4 oral swabs.

FCoV RT-qPCR results, also according to species, facility, sex, age, and clinical status, are reported in Table 3. Positive RT-qPCR results were found in 3 out of 38 animals (7.9%), represented by three 1-month-old tiger cubs. FCoV-positive tiger cubs came from the same litter, which was composed of one female and two males. Of these tiger cubs, one male showed neurological signs suggestive of vestibular dysfunction. Positive samples consisted of two rectal and three oral swabs, with Ct values ranging from 34.5 to 38.9 and relative FCoV copy numbers ranging from 30.2 to 1.2 (Table 4). Rectal swabs showed positive results in one animal only, both at the first and second samplings, whereas negative results were obtained at the third sampling. Oral swabs showed positive results in all three animals. The dam tested FCoV negative 55 days after parturition.

Based on molecular tests, laboratory analyses and follow up, none of the unhealthy animals with clinical signs suggestive of FIP were actually affected by this disease. More precisely, two animals showed abdominal effusion and one animal showed central nervous system signs suggestive of FIP [18], but the diagnostic procedures performed within the VTH showed that one snow leopard was affected by clostridiosis, as demonstrated by the analysis of the abdominal effusion, one cheetah had lymphoma which was diagnosed via cytology and flow cytometry, and one tiger cub had transient neurological signs that completely recovered after corticosteroid therapy. Therefore, in agreement with the zoological facility veterinarians, no other tests were performed to achieve a final diagnosis. Three other animals showed clinical signs suggestive of diseases other than FIP, such as a bone fracture, a soft tissue sarcoma located in the scapular region associated with chronic kidney disease, as demonstrated by serum biochemistry, and corneal ulcers, which were likely due to nutritional deficiencies. The main clinical–pathological findings from these animals are summarized in Table 5.

## 4. Discussion

FCoV is a ubiquitous virus of domestic cats, and a small proportion of FCoV-infected cats develop FIP, a disease characterized by a high mortality rate which is reported worldwide [5]. FCoV-associated FIP cases have also been reported in non-domestic members of the Felidae family [2,6]. FIP cases in non-domestic felids are of concern, particularly in endangered populations, and the need for surveys of FCoV prevalence in these animals has been highlighted [2]. Due to the lack of information on FCoV prevalence in captive non-domestic felids in Europe, our study focused on RT-qPCR detection of FCoV in non-domestic felids from zoological facilities in Northern Italy. Given that FCoV infection has been previously reported in healthy animals [2,10], non-domestic felids from this study were sampled regardless of their health status.

The overall 7.9% FCoV molecular positivity and the FCoV prevalences ranging from 0% to 60% according to the zoological facility observed in our study are in accordance with previous studies reporting different prevalence worldwide in non-domestic felids, showing a FCoV molecular prevalence ranging from 0% to 32% in zoological facilities in Southern Brazil and the USA, respectively [10,12]. The presence of FCoV infection in tigers observed in this study is in agreement with previous reports showing FCoV presence and FIP cases in this animal species [2,10], and it is likely explained by the higher number of tigers investigated in our study compared to other species of non-domestic felids.

The low FCoV copy numbers observed in the positive samples from tiger cubs are in agreement with previous reports of low viral load in samples from healthy FCoV-infected cats [29,31]. FCoV infection in the absence of FIP cases in these tiger cubs was expected, and is in accordance with previous reports showing FCoV infection in healthy tigers [10,32]. Even if neurological signs can be due to the dry form of FIP, the clinical outcome of the FCoV positive tiger cub that recovered soon after the treatment is not suggestive of FIP [18]. Moreover, FIP cases have not been identified as cause of mortality in Italian zoos between 2004 and 2015, and FIP has not been frequently reported in non-domestic felids housed in zoological facilities worldwide [2,3].

Our study focused on the identification of viral shedding by RT-qPCR using fecal samples or rectal swabs that are known to be the samples of choice for FCoV detection and that have also been used for the identification of carrier animals among non-domestic felids [16]. The use of rectal swabs allowing the identification of FCoV in only one out of the three FCoV-positive animals was not surprising, and confirms the need for adequate multiple fecal samplings for the identification of all shedders, including animals that shed virus intermittently or rarely, as previously reported for domestic and non-domestic felids [16,33].

Identification of FCoV positivity in all three tiger cubs using oral swabs, especially in the two tiger cubs with negative RT-qPCR results on rectal swabs, was not expected, and contamination by maternal grooming may not be excluded. In cats, the use of saliva is not recommended to detect FCoV [33], even if the presence of FCoV in the oral cavity has been rarely investigated. Stoddard and colleagues (1988) reported that during the early stages of experimental infection in cats, FCoV presence was initially observed only in oropharyngeal swabs, before shedding of the virus occurred in feces, followed by FCoV presence in both oropharyngeal swabs and feces until the end of the experimental study [34]. Further investigations are suggested to study the early phase of FCoV infection in non-domestic felids to understand shedding patterns.

The source of infection for tiger cubs was not identified, confirming previous studies reporting difficulties in the identification of the source of FCoV infection in non-domestic felids [2]. In cats, infection of kittens is commonly caused by virus shedding by the queen, or by high or persistent shedders in the cattery [15]. Regarding the presence of shedders as source of infection, previous reports consider cats an unlikely source of FCoV infection in non-domestic felids, and suggest that FCoV transmission may be through direct or indirect contact with conspecific or other non-domestic felids [2]. Direct contact of the cubs was with the dam. The dam was apparently healthy during pregnancy and after the birth of the cubs and it was not possible to establish if the dam was the likely source of infection, since the dam was tested only once. The negative result of the dam based on a single time point sample may not identify FCoV shedding, and needs to be confirmed by consecutive fecal samples analysis [16].

FCoV risk factors were not analyzed in our study due to the limited number of samples that did not allow statistical analysis to be performed. Based on our results showing a higher presence of FCoV in young animals compared to adults, it would be interesting to further investigate age as a risk factor for FCoV infection in non-domestic felids, as previously reported for FIP in domestic cats [15,18]. The FCoV presence detected only in one out of three sampled facilities leading to a highly variable prevalence in the different zoological facilities, as observed in our study, could be due to different management or biosecurity practices carried out in the facilities or to the introduction of an undetected FCoV shedder, since FCoV is not routinely tested in non-domestic felids. Therefore, further investigations are needed to understand the epidemiology of FCoV infection among non-domestic felids and to identify possible risk factors.

The low viral load in positive samples did not allow genetic characterization of FCoV using classical RT-PCR methods and sequencing. Future studies should also imply diagnostic strategies to genetically characterize FCoV in non-domestic felids in order to understand phylogenetic correlations among FCoV strains.

## 5. Conclusions

In conclusion, the presence of FCoV in non-domestic felids from zoological facilities was confirmed in Northern Italy and highlights the need for continuous FCoV infection monitoring to prevent FCoV introduction into a naïve group of animals, which may lead to devastating effects [2]. As previously suggested [14], confirmation of FCoV prevalence may be recommended using a combination of molecular techniques and serology, as future analysis should also include data on seroprevalence in Italian zoological facilities.

One of the main limitations of this study is the low sample size and the limited number of included zoological facilities. However, it must be taken into account that the number of these species in the Italian territory is low by itself, as non-domestic felids can only be kept by authorized facilities and sampling may not be easy to perform, especially for blood samples that are required for serological studies.

Given the ease of collecting fecal samples, future prospective studies should be carried out, including broader fields and larger sample numbers of non-domestic felids in order to gain a deeper knowledge of FCoV epidemiology and shedder status within the captive non-domestic felid populations and, as a practical aspect, to define strategies to prevent spread of the virus.

## Figures and Tables

**Table 1:** Characterization of non-domestic felids analyzed in this study.

Variable	Category	Total	Species						
			**Tiger**	**Lion**	**Cheetah**	**Snow Leopard**	**Leopard**	**Clouded Leopard**	**Mountain Lion**
Zoo	1	14 (37%)	7	7	0	0	0	0	0
	2	19 (50%)	8	1	4	2	2	1	1
	3	5 (13%)	4	0	0	1	0	0	0
Sex	Male	19 (50%)	10	4	1	2	1	1	0
	Female	19 (50%)	9	4	3	1	1	0	1
Age	≤2 years	5 (13%)	3	0	0	1	1	0	0
	>2 years	33 (87%)	16	8	4	2	1	1	1
Health status	Clinically healthy	32 (84%)	16	8	3	2	1	1	1
	Presence of clinical signs suggestive of FIP	3 (8%)	1	0	1	1	0	0	0
	Other diseases	3 (8%)	2	0	0	0	1	0	0
Total		38	19	8	4	3	2	1	1

**Table 2:** Number and type of specimens analyzed in this study.

Species	Specimen						
	Fecal/Rectal Single Sampling	Fecal/Rectal Double Sampling	Fecal/Rectal Triple Sampling	Blood	Oral Swab Single Sampling	Oral Swab Double Sampling	Abdominal Effusion
Tiger	13	3	3	1	2	1	0
Lion	7	1	0	0	0	0	0
Cheetah	4	0	0	1	0	0	1
Snow leopard	2	1	0	1	0	0	1
Leopard	2	0	0	1	0	0	0
Clouded leopard	0	1	0	0	0	0	0
Mountain lion	1	0	0	0	0	0	0
Total	29	6	3	4	2	1	2

**Table 3:** Number and percentage of non-domestic felids with FCoV RT-qPCR positive results according to species, facility, sex, age, and health status.

Variable	Category	No.	FCoV Positive
Species	Tiger	19	3 (15.8%)
	Lion	8	0
	Cheetah	4	0
	Snow leopard	3	0
	Leopard	2	0
	Clouded leopard	1	0
	Mountain lion	1	0
Zoological facility	1	14	0
	2	19	0
	3	5	3 (60%)
Sex	Male	19	2 (10%)
	Female	19	1 (5%)
Age category	≤2 years	5	3 (60%)
	>2 years	33	0
Health status	Clinically healthy	32	2 (6%)
	Presence of clinical signs suggestive of FIP	3	1 (33%)
	Other diseases	3	0

**Table 4:** RT-qPCR results in tiger cubs and dam samples.

Animal ID	Sampling Days after Birth	Rectal Swab *	Oral Swab *
Cub 1	21	Negative	Positive (34.9; 21.5)
	34	Negative	Negative
	55	Negative	NC
Cub 2	21	Positive (34.5; 30.2)	NC
	34	Positive (36.8; 5.3)	Positive (37.5; 3.4)
	55	Negative	NC
Cub 3	21	Negative	NC
	34	Negative	Positive (38.9; 1.2)
	55	Negative	NC
Dam	55	Negative (feces)	NC

* Cycle threshold values; FCoV copy number in parentheses. NC—not collected.

**Table 5:** Clinical features, final diagnosis, and FCoV status of unhealthy animals.

Species	Clinical Findings	FCoV qPCR	Final Diagnosis
Tiger	Neoformation in the scapular region	Negative	Soft tissue sarcoma, chronic kidney disease
Cheetah	Abdominal effusion, lymph nodes enlargement	Negative	Multicentric B lymphoma
Leopard	Lameness	Negative	Tibial fracture
Snow leopard	Abdominal effusion, sudden death	Negative	*Clostridiosis*
Tiger	Ocular ulcers	Negative	Undetermined
Tiger	Transient neurological signs	Positive	Undetermined

## Data Availability

The data that support the findings of this study are available from the corresponding author upon reasonable request.
